# Translation, cultural adaptation, and validation of the NOSE-Perf scale to Brazilian Portuguese

**DOI:** 10.1016/j.bjorl.2024.101442

**Published:** 2024-05-16

**Authors:** Fabio Portella Gazmenga, Mariana Dalbo Contrera Toro, Fabio Lau, Arthur Jose Roque Cruz, Elaine Costa, Michael J. Marino, Eulalia Sakano

**Affiliations:** a Universidade Estadual de Campinas (Unicamp), Departamento de Otorrinolaringologia e Cirurgia de Cabeça e Pescoço, Campinas, SP, Brazil; b Mayo Clinic, Department of Otolaryngology — Head and Neck Surgery, Phoenix, USA

**Keywords:** Nasal septal perforation, Quality of life, Outcomes, Questionnaire

## Abstract

**Objective:**

To perform the translation, cultural adaptation, and validation of the NOSE-Perf Scale to Brazilian Portuguese.

**Methods:**

This study was divided into two stages. In the first stage, the questionnaire was submitted for translation and cultural adaptation, following the guidelines recommended by the ISPOR Task Force (International Society for Pharmacoeconomics and Outcomes Research). Then, the Brazilian Portuguese version of the NOSE-Perf scale was applied to a group with septal perforation and a control group. The group with perforation answered the questionnaire again after one month. Internal consistency, test-retest reliability, and discriminant validity were assessed.

**Results:**

The Brazilian Portuguese version of the NOSE-Perf scale was applied to 32 participants, 16 from the group with septal perforations and 16 controls. The instrument obtained high internal consistency, with Cronbach's alpha scores of 0.986. High reliability was also obtained, with Spearman's correlation coefficient of 0.996 (*p* < 0.001) and the intraclass correlation coefficient of 0.965 with a 95% Confidence Interval (95% CI) of 0.886‒0.988. The NSP group obtained a mean total score of 13.8 ± 12.6 and the control group a score of 2.3 ± 1.8, with a statistical difference between the groups (*p* < 0.001), demonstrating good discriminant validity.

**Conclusion:**

The Brazilian Portuguese version of the NOSE-Perf scale is a reliable and valid instrument for measuring symptoms in patients with nasal septum perforations.

**Level of evidence:**

Level 2—Oxford Centre for Evidence-Based Medicine 2011 Levels of Evidence.

## Highlights

• NOSE-Perf scale quantifies symptoms resulting from nasal septal perforation.
• The Portuguese version of the NOSE-perf scale showed internal consistency.
• The NOSE-Perf scale translation showed good reliability parameters.
• The proposed instrument is valid for measuring nasal septal perforation symptoms.

## Introduction

Nasal septal perforation (NSP) is a pathological condition that establishes communication between the two nasal cavities due to a septal defect. It emerges from a range of pathologies, including traumatic, iatrogenic, drug use, and various inflammatory, rheumatic, infectious, and neoplastic diseases.^1^, ^2^

Its clinical repercussions are very diverse, ranging from asymptomatic patients to symptoms that significantly disrupt the Quality of Life (QoL), such as nasal obstruction, whistling, crusting, epistaxis, pain, dryness, and nasal deformity.^3^, ^4^, ^5^, ^6^ Thus, its treatment is something challenging for rhinologists and can range from conservative measures, such as nasal irrigation and emollients, to the use of septal buttons or surgical interventions, with a variety of techniques described in the literature.^2^, ^7^, ^8^

A fundamental part of the management of any condition is to understand the impact of each symptom on the patient's QoL. QoL assessment instruments are important not only to better understand the disease but also to standardize the way of reporting treatment outcomes. To this end, the NOSE-Perf scale was recently described as a validated disease-specific symptom score for NSP.^9^ However, it is currently only available in English, and there are no similar instruments validated for the Brazilian Portuguese-speaking population.

The aim of this study was to perform the Translation, Cultural Adaptation, and Validation of the NOSE-Perf Scale to Brazilian Portuguese.

## Methods

This study was developed in two stages. The first consisted of the translation and cultural adaptation of the original questionnaire into Brazilian Portuguese and the second of its prospective validation.

### Ethical considerations

This study was authorized by the Ethics Committee of the University, under registration number 59360722.0.0000.5404. Informed consent was obtained from all participants.

### Translation and cultural adaptation

Translation and cultural adaptation followed the recommendations of the ISPOR Task Force^10^ and was divided into 7 steps, as follows.

1) Preparation: Authorization was initially obtained from the author of the original questionnaire to conduct the study.
2) Forward Translation: The questionnaire was translated into Portuguese independently by two Brazilian rhinologists fluent in English.
3) Reconciliation: The two versions were evaluated by a committee of three rhinologists and reconciled into a single version.
4) Back translation: From this unified version, a back translation into English was performed by a native speaker of English who is also fluent in Portuguese.
5) Back-translation review and harmonization: The back-translated version was compared to the original questionnaire. All translations performed were discussed and analyzed by all those involved in the previous steps, resulting in a preliminary version of the questionnaire.
6) Cognitive debriefing: This preliminary version was applied to a representative group of 10 patients with septal perforations. Each patient answered the questionnaire autonomously, and afterward, the whole text was discussed with a rhinologist to review comprehension, clarity of questions, and possible suggestions.
7) Final revision and final report: The findings of the cognitive debriefing were considered to improve the cultural adaptation, and the questionnaire was again revised by the committee of rhinologists, generating the final version of the NOSE-Perf translated into Brazilian Portuguese. The final version and the report were sent to the author of the original questionnaire for his approval before starting the validation.

### Participants

We included 16 consecutive patients from the rhinology outpatient clinic with the diagnosis of septal perforation were, and 16 participants without septal perforation and sinonasal comorbidities for the control group.

For the group with septal perforations, the inclusion criteria were patients older than 18 years, literate, with a diagnosis of septal perforation, without other sinonasal comorbidities, and who did not undergo any intervention between the first and second answering of the questionnaire. Exclusion criteria: active nasosinusal disease, previous skull base surgery, history of sinonasal malignancy, previous head and neck radiation therapy, craniofacial syndrome, current use of intranasal drugs, cleft palate, or previous cleft palate repair.

### Application of the final version of the Questionnaire

Participants were instructed to answer the final version of the NOSE-Perf in Portuguese by themselves. A physician was always present, but without intervening. In addition, to assess clarity, the patients were asked to classify the question as “easy to understand”, “difficult to understand”, and “did not understand” for each question.

The control group answered the questionnaire only once to perform validation. The perforation group answered the questionnaire again after one month to assess the stability of the responses using test-retest reliability.

### Statistical analyses

The sample size was calculated according to Bonett’s sample size requirements^11^ using the Arifin sample size calculator (https://wnarifin.github.io/ssc_web.html) and based on the estimate of the questionnaire being filled out by the same individual on two different occasions, hoping to find an ICC of 0.9 a 0.1 precision, and an alpha value of 0.05. This resulted in a sample size of 15 patients, taking into account that, although unexpected, we could have a dropout, the final sample size was calculated to be 16, similar to Camina et al.^12^ The same number of patients was chosen to form the control group.

IBM® SPSS® software version 29.0 was used for analysis. Qualitative variables are presented in absolute and relative frequencies. The quantitative variables' means, standard deviations, and minimum and maximum amplitudes were determined. Sociodemographic and clinical characteristics were analyzed using the Chi-Squared, Fisher Exact, and independent sample *t*-tests. To assess internal consistency, Cronbach’s alpha coefficient was calculated, with values between 0.7 and 0.95 considered acceptable. Test–retest reliability was analyzed using Spearman’s correlation coefficient. The Intraclass Correlation Coefficient (ICC) with absolute-agreement, 2-way mixed-effects model was determined by correlating the measurements obtained in the first and the second visit.

For discriminant validity, the total scores of the quiz of the two groups were compared. Data were analyzed using a nonparametric Mann–Whitney *U* test. A *p*-value of < 0.05 was considered significant.

## Results

### Patient characteristics

Table 1 summarizes the patient’ characteristics. There was no statistical difference between the groups regarding age, gender, and education.

**Table 1:** Patients’ characteristics.

	NSP group (n = 16)	Control group (n = 16)	*p*
**Mean age (SD)**	51.9 (SD = 17.7)	47.6 (SD = 16.1)	**0.469**^a^
**Female gender**	11 (68.8%)	9 (56.2%)	**0.533**^b^
**Scholarity**			**0.716**^c^
Incomplete elementary school	6 (37.5%)	4 (25%)	
Complete elementary school	3 (18.8%)	4 (25%)	
High school	5 (31.3%)	6 (37.5%)	
College	2 (12.5%)	2 (12.5%)	
**Septal Perforation Etiology**	GPA Sequel: 6 (18.8%)		
	Idiopathic: 5 (15.6%)		
	Iatrogenic: 2 (6.3%)		
	Leishmaniosis Sequel: 1 (3.1%)		
	Trauma: 1 (3.1%)		
	AIFR Sequel: 1 (3.1%)		

GPA, means granulomatosis with polyangiitis; NSP, nasal septal perforation; AIFR, acute invasive fungal rhinosinusitis; SD, standard deviation.

a *t*-test.

b Chi-Squared test.

c Fisher exact test.

In our sample, there were seven different etiologies for septal perforations. In six patients, the NSP was a late sequela of granulomatosis with polyangiitis, all with stable disease and no other sinonasal repercussions, 5 idiopathic cases, 2 patients with iatrogenic perforation after septoplasty, 1 case with late sequelae of Leishmaniasis, without active disease or other sinonasal repercussions, 1 case after facial trauma and 1 patient with sequelae of acute invasive fungal rhinosinusitis, with the cured disease.

### Questionnaire

#### Translation and cultural adaptation

**Figure 1:**
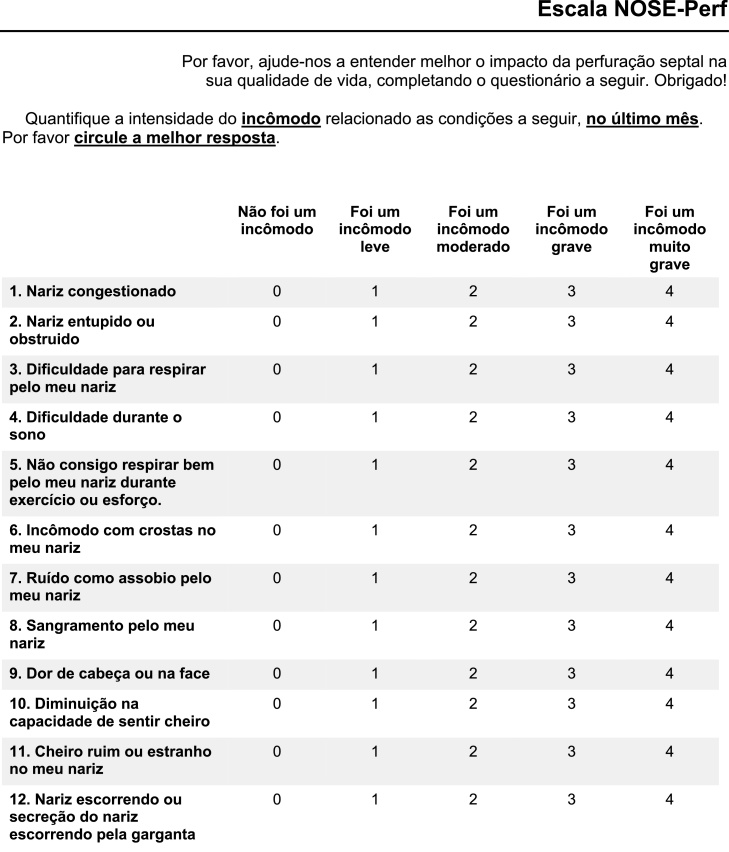
Final version of NOSE-Perf scale for Brazilian Portuguese.

The final version of the NOSE-Perf questionnaire translated into Brazilian Portuguese is shown in Fig. 1.

**Figure 2:**
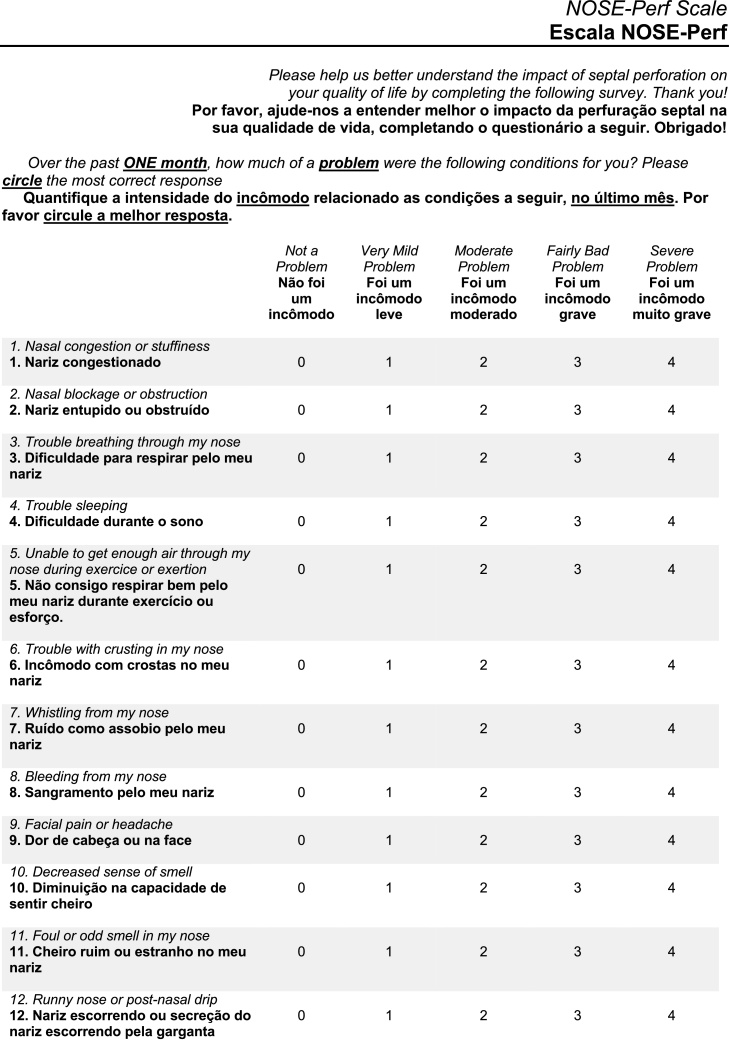
Comparison between the original NOSE-Perf scale and the version translated into Portuguese. The original sentences are in italics and their respective translations are in bold.

Fig. 2 compares the original NOSE-Perf sentences in English with their respective translations into Portuguese.

#### Clarity

**Figure 3:**
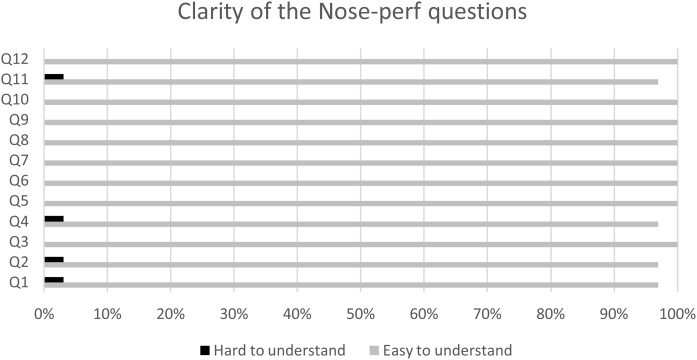
Clarity of the Nose-Perf questions translated to Portuguese. Participants were asked if the question was easy to understand, hard to understand, or if they did not understand.

Fig. 3 illustrates how the 32 participants rated the clarity of the questions. For questions 1, 2, 4, and 11, only one patient rated them as “difficult to understand”. For the other questions, all 32 patients rated them as “easy to understand”. None of the participants rated a question as “I did not understand”.

#### Discriminant validity and questionnaire scores

Table 2 shows the total score of the scale for the two groups evaluated, with their mean and minimum amplitudes. The NSP group obtained a mean total score of 13.8 ± 12.6. The control group obtained a mean total score of 2.3 ± 1.8. There was a statistical difference between the groups, with *p* < 0.001, demonstrating good discriminant validity.

**Table 2:** NOSE-Perf-scale total score for both groups, showing mean, minimum and maximum score and standard deviation.

	n	Mean‒NP	Min‒Max	SD	*p*^a^
**NSP Group**	16	13.8	0‒35	12.6	<0.001
**Control Group**	16	2.3	0‒5	1.8	

a Mann–Whitney *U* test.

Table 3 details the mean scores for each of the questions and the two responses from the NSP group. For these patients, crusting was the main problem, with a score of 1.87 and 1.69 (first and second visit, respectively), while whistling had the lowest score (0.81 and 0.69). The other symptoms assessed had similar scores. As for the control group, the most reported symptom was facial pain, with a score of 0.69, while whistling and epistaxis were not scored by any participant. Table 4 demonstrates the means scores, SD value, and comparison between the NSP group and control group. It also describes the item-total correlation for each question, which showed a good overall correlation, with all questions above 0.5.

**Table 3:** Comparison of mean scores for each question for the nasal septal perforation (NSP) Group in their first and second visits.

	NSP Group 1st visit	SD	NSP Group 2nd visit	SD	*p*-Value^a^
**Question 1**	1	1.21	1.06	1.29	0.919
**Question 2**	1.19	1.38	1.19	1.38	1
**Question 3**	1.13	1.46	1.25	1.48	0.758
**Question 4**	1.13	1.46	1.19	1.47	0.950
**Question 5**	1	1.37	1.25	1.24	0.446
**Question 6**	1.87	1.37	1.69	1.4	0.685
**Question 7**	0.81	1.17	0.69	1.08	0.862
**Question 8**	1	1.37	0.94	1.24	0.951
**Question 9**	1.5	1.5	1.19	1.17	0.625
**Question 10**	1	1.37	0.81	1.22	0.802
**Question 11**	1.06	1.44	0.94	1.29	0.793
**Question 12**	1.13	1.54	0.88	1.59	0.442

SD, standard deviation.

a Mann–Whitney *U* test.

**Table 4:** Comparison between each question for the nasal septal perforation group (NSP) and Control Group (CG). Item-total correlation for each question for the NSP group.

	Group	Mean	Standard deviation	Item-total correlation^b^	*p*-Value^a^
**q1**	NSP	1.00	1.211	*0.862*	*0.118*
	CG	0.31	0.479		
**q2**	NSP	1.19	1.377	*0.866*	***0.032***
	CG	0.25	0.447		
**q3**	NSP	1.13	1.455	*0.898*	*0.108*
	CG	0.25	0.447		
**q4**	NSP	1.13	1.455	*0.881*	*0.124*
	CG	0.31	0.602		
**q5**	NSP	1.00	1.366	*0.897*	***0.032***
	CG	0.13	0.342		
**q6**	NSP	1.88	1.360	*0.886*	***<0.001***
	CG	0.13	0.342		
**q7**	NSP	0.81	1.167	*0.877*	***0.008***
	CG	0.00	0.000		
**q8**	NSP	1.00	1.366	*0.530*	***0.004***
	CG	0.00	0.000		
**q9**	NSP	1.50	1.506	*0.566*	*0.101*
	CG	0.69	1.014		
**q10**	NSP	1.00	1.366	*0.663*	***0.032***
	CG	0.13	0.342		
**q11**	NSP	1.06	1.436	*0.806*	***0.025***
	CG	0.06	0.250		
**q12**	NSP	1.13	1.544	*0.720*	***0.012***
	CG	0.06	0.250		

a Pearson correlation.

b Mann–Whitney *U* test.

#### Internal consistency and test-retest reliability

Cronbach’s alpha score for the Nose-Perf score was 0.996, indicating high internal consistency.

**Figure 4:**
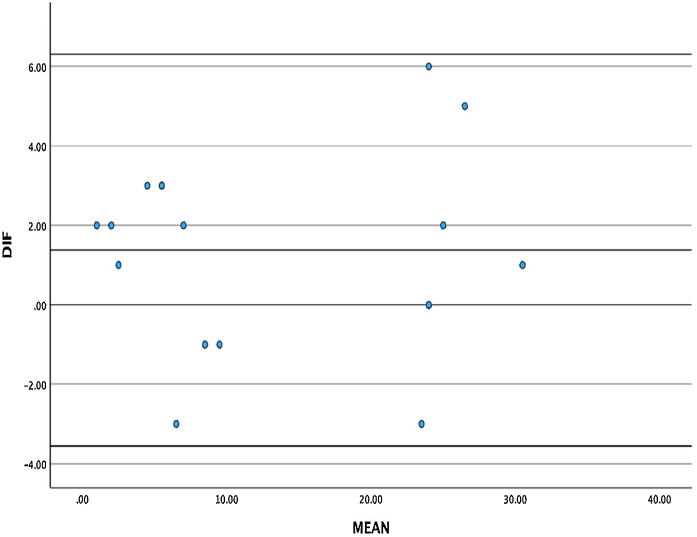
Bland and Altman graphic representation of reproducibility. DIF, The difference between the total scores from the first and second visits. MEAN, Mean values of the questionnaire scores. The darker lines represent Standard deviation values.

Spearman’s correlation coefficient was 0.994 (*p* < 0.001), indicating the high reliability of repeated measures. The intraclass correlation coefficient was 0.992 with a 95% Confidence Interval (95% CI) of 0.978‒0.997, indicating high reliability. Fig. 4 represents the Bland-Altman plot of the difference between the questionnaire scores in the first and second visits, indicating that their mean values are within two standard deviations (1.96 × SD: Standard Deviation), demonstrating agreement between both visits' answers.

## Discussion

This study performed the translation, cultural adaptation, and validation of the Nose-Perf scale^9^ into Brazilian Portuguese, following the Principles of Good Practice recommended by the ISPOR taskforce,^10^ ensuring equivalence between the original version and the one adapted to the Brazilian culture.

The participant’s level of education was representative of the non-illiterate Brazilian population, according to 2022 data from the Brazilian Institute of Geography and Statistics (IBGE-https://censo2022.ibge.gov.br/panorama/). Despite the low education level in general, we obtained excellent understanding and clarity in all questions.

NSP can lead to significant impairment in patients’ quality of life. Using the SNOT-22, Leong and Webb^13^ assessed the impact of NSP symptoms on QoL, comparing scores with those of patients with CRS. Both scores were high, indicating great impairment in QoL and, despite being higher in the CRS group, there was no significant difference between them. Subsequently, Khong and Leong^14^ reported that SNOT-22 scores of patients with NSP were comparable to other diseases with a major impact on QoL, such as recalcitrant chronic rhinosinusitis, empty nose syndrome, and neurogenic facial pain syndromes. In both studies, the authors highlighted the limitation of using a non-specific instrument to assess NSP symptoms and the importance of creating one that addresses specific symptoms of the disease, such as nasal crusting, epistaxis, and whistling noise.

In this context, the NOSE-Perf scale was developed, a disease-specific instrument to assess and quantify symptoms resulting from septal perforation.^9^ After its validation and publication, the same group used this instrument to evaluate NSP baseline symptoms in 202 patients.^6^ The most prevalent symptom was crusting, followed by congestion, breathing difficulty, obstruction, rhinorrhea, epistaxis, and whistling. In our study, despite crusting was also the most common, the prevalence of other symptoms was variable, differing from those reported by Taylor et al.^6^

Our NSP patients were also less symptomatic, with a mean total NOSE-Perf score of 13.8 compared to 23.7 in the aforementioned study.^6^ Although NSP is a condition that potentially affects QoL, our sample reported a minor impact, with individual average for each of the 12 items not reaching 2 out of a possible 4 points (Table 3). Compared to previous studies that reported symptoms related to NSP, there is also a significant variability in symptom prevalence,^15^, ^16^, ^17^, ^18^, ^19^, ^20^, ^21^ thus it is expected their respective impacts on QoL would also vary.

NSP clinical manifestations are related to the size and location of the NSP.^3^, ^14^, ^22^ Anterior NSPs are the most frequent and usually more symptomatic, while posterior or superior NSPs are often asymptomatic and more commonly caused by systemic diseases.^3^, ^22^, ^23^ Therefore, these differences between published studies can be explained in part by different samples regarding the etiology and location of NSP, in addition to ethnically and regionally different populations.

Evaluating the etiologies of NSP, we found a high prevalence of sequelae of rheumatological diseases and idiopathic cases, and a relatively small percentage of cases secondary to surgical procedure, a frequent cause of NSP.^2^, ^4^ This can be explained by the study being carried out in a tertiary university reference center, with a low volume of septoplasties and a high prevalence of rare diseases and complex cases. However, discussion regarding etiology is limited by the small number of patients participating in this study.

This study highlights the clinical heterogeneity of the disease and the importance of having an instrument that explicitly evaluates each symptom so that it is possible to understand each case better, making the treatment more accurate and personalized.

In practical terms, the questionnaire proved easy and quick to complete and may also give patients greater insight into the severity of their symptoms and the outcomes of medical interventions.

The Brazilian Portuguese version of the NOSE-Perf scale showed excellent clarity and understanding by the participants, good reliability, and proved to be valid for use in the Brazilian population. This instrument may contribute to standardizing the documentation of NSP symptoms among Brazilian physicians and provide a deeper understanding of this disease’s impact on the Brazilian population’s QoL. It may also be used to report treatment outcomes and enable comparison with other international studies using the NOSE-Perf scale.

This study had the limitation of producing an instrument to be self-applied, limiting its use in the illiterate population. Another challenge of any cultural adaptation of an instrument for the Brazilian population is the fact that it is a large country with culturally heterogeneous regions.

## Conclusion

The Brazilian version of the NOSE-Perf scale is a reliable and valid instrument for measuring symptoms in patients with nasal septal perforations.

## Authors’ information and contributions

Mariana Dalbo Contrera Toro: Contributed to drafting the manuscript.

Fabio Lau: Contributed to drafting the manuscript.

Arthur Jose Roque Cruz: Contributed to drafting the manuscript.

Elaine Costa: Contributed to drafting the manuscript.

Michael J. Marino: Contributed to drafting and revising the manuscript critically for important intellectual content.

Eulalia Sakano: Contributed to the conception and design; and acquisition, analysis, and interpretation of data.

## Funding

None to declare.

## Conflicts of interest

The authors declare no conflicts of interest.
